# An Adjustable Dark-Field Acoustic-Resolution Photoacoustic Imaging System with Fiber Bundle-Based Illumination

**DOI:** 10.3390/bios11080262

**Published:** 2021-08-03

**Authors:** Yuhling Wang, De-Fu Jhang, Tsung-Sheng Chu, Chia-Hui Tsao, Chia-Hua Tsai, Chiung-Cheng Chuang, Tzong-Rong Ger, Li-Tzong Chen, Wun-Shaing Wayne Chang, Lun-De Liao

**Affiliations:** 1 Institute of Biomedical Engineering and Nanomedicine, National Health Research Institutes, Miaoli County 35053, Taiwan; yuhlingwang@nhri.edu.tw (Y.W.); g10702501@cycu.edu.tw (D.-F.J.); tsungsheng_chu@cycu.org.tw (T.-S.C.); tsaochiahui@nhri.edu.tw (C.-H.T.); vanessatsai@nhri.edu.tw (C.-H.T.); 2 Department of Biomedical Engineering, College of Engineering, Chung Yuan Christian University, Taoyuan City 32023, Taiwan; cheng965@cycu.edu.tw (C.-C.C.); sunbow@cycu.edu.tw (T.-R.G.); 3 National Institute of Cancer Research, National Health Research Institutes, Miaoli County 35053, Taiwan; 4 Kaohsiung Medical University Hospital, Kaohsiung Medical University, Kaohsiung City 80708, Taiwan

**Keywords:** fiber-bundle-based illumination, hemoglobin oxygenation saturation, in vivo imaging, photoacoustic (PA)

## Abstract

Photoacoustic (PA) imaging has become one of the major imaging methods because of its ability to record structural information and its high spatial resolution in biological tissues. Current commercialized PA imaging instruments are limited to varying degrees by their bulky size (i.e., the laser or scanning stage) or their use of complex optical components for light delivery. Here, we present a robust acoustic-resolution PA imaging system that consists of four adjustable optical fibers placed 90° apart around a 50 MHz high-frequency ultrasound (US) transducer. In the compact design concept of the PA probe, the relative illumination parameters (i.e., angles and fiber size) can be adjusted to fit different imaging applications in a single setting. Moreover, this design concept involves a user interface built in MATLAB. We first assessed the performance of our imaging system using in vitro phantom experiments. We further demonstrated the in vivo performance of the developed system in imaging (1) rat ear vasculature, (2) real-time cortical hemodynamic changes in the superior sagittal sinus (SSS) during left-forepaw electrical stimulation, and (3) real-time cerebral indocyanine green (ICG) dynamics in rats. Collectively, this alignment-free design concept of a compact PA probe without bulky optical lens systems is intended to satisfy the diverse needs in preclinical PA imaging studies.

## 1. Introduction

In medical research, the use of optical imaging techniques is of particular interest because the intrinsic optical contrast found in in vivo systems can be used instead of having to inject contrast agents [1]. In addition, the nonionizing radiation used in optical imaging techniques is safer for use in humans [2]. However, the strong light scattering in pure optical imaging modalities results in poor spatial resolution and shallow penetration depth [3,4,5]. An example is diffuse optical tomography (DOT), in which the scattering behavior of photons in tissue is modeled to reconstruct images [6]. The penetration depth for DOT is only a few millimeters [7]. As the spatial resolution is approximately 1/5th the imaging depth, the DOT technique additionally suffers from poor spatial resolution [3,6]. The maximum penetration depth for optical microscopy (i.e., confocal microscopy and two-photon microscopy) using ballistic or quasi-ballistic photons is typically limited to one optical transport mean free path (~1 mm) [8]. This fundamental light diffusion issue is an obstacle to the widespread preclinical and clinical application of pure optical imaging techniques [3,7].

The principle of photoacoustic (PA) imaging is based on optical absorption, and this imaging is characterized by deep tissue penetration and multiscale spatial resolution [9]. In PA imaging, ultrasound (US) imaging is combined with intrinsic optical absorption [10]. A pulsed laser wavelength-tunable from the visible to near-infrared (NIR) is selected to deliver laser energy to biological samples [11]. The optical absorption of the biological sample induces PA waves via the thermoelastic effect. Then, the optical absorption distribution in the biological sample can be reconstructed from the PA signal detected by a designated US transducer [10]. Similar to other optical imaging modalities, such as DOT and confocal microscopy, PA imaging has an intrinsic contrast ability [3,8]. However, the PA technique has the advantage of deeper penetration depth (up to 5 cm) through the use of ultrasonic spatial resolution [10]. Intrinsic absorptive molecules that can be detected by the PA technique also provide good contrast for the in vivo imaging of living tissue [4,12]. Moreover, based on the intrinsic optical contrast of biological tissues (i.e., blood or melanin) [10], the PA technique can provide structural (i.e., angiogenesis) and functional (i.e., hemoglobin oxygen saturation and total hemoglobin concentration) information [11,13,14].

Thus, a reflection-mode dark-field PA microscopy (PAM) imaging technique using a high-frequency US transducer (i.e., >20 MHz) was developed that can track blood oxygenation dynamics in the mouse brain in vivo under global hypoxic and hyperoxic conditions [15]. Recently, we published several studies showing that functional PAM (fPAM) is an ideal tool for in vivo evaluation of the changes in functional cerebral blood volume (CBV) and hemoglobin oxygen saturation (SO_2_) in normal rat brains [16,17,18] or in disease models [19,20,21]. Additionally, PAM has been extended to theranostic applications [22], such as treatment intervention [23], chemotherapy [24], and imaging-guided photothermal therapy [25,26]. Researchers have also explored PA imaging agents as contrast enhancement agents [27,28] or drug carriers [25,29], where drug release is triggered by the heat generated by the agent upon laser irradiation. Overall, fPAM has been used in increasing numbers of preclinical applications in recent years [30]. However, the bulkiness of the associated equipment and lack of a simplified user interface prevent the wide use of fPAM technology in clinical imaging [10,30]. An additional challenge is fiber damage at the tip surface, which is caused by the high peak power density generated when focusing and coupling light into the fiber during the delivery of high-energy laser pulses [5]. Hence, the output energy in optical fiber delivery must be limited, which restricts the illumination area and penetration depth of PA technology.

In this study, we report a combined US and acoustic-resolution PA imaging system with fiber bundle-based illumination. The developed system is fully programmable using MATLAB-based software. To allow the user to make selections based on the application of interest, the US system is designed to accommodate transducers of different types/frequencies. Most importantly, a new probe concept using this fiber bundle-based illumination system is developed, which externally couples light energy to the imaging zone of a minimized optical parametric oscillator (OPO) laser and is fixed to the transducer using a three-dimensional (3D)-printed holder. The system is more compact and easier to set-up because of the lack of optical lens systems. Data acquisition, image processing, and control of the laser or scanning stage were performed using a MATLAB-based software platform. To validate the developed US/PA system in vitro, the signal-to-noise ratios (SNRs) in PA images of different concentrations of blue ink in a tube phantom placed at a 9 mm depth were obtained. Next, we tested the in vivo functional ability of the developed US/PA system to image (1) rat ear vasculature, (2) real-time cortical hemodynamic changes in the superior sagittal sinus (SSS) during left-forepaw electrical stimulation [31], and (3) real-time dynamics of cerebral indocyanine green (ICG) in rats. Collectively, this alignment-free design concept of a compact PA probe is intended to satisfy the diverse needs of researchers in preclinical PA studies.

## 2. Materials and Methods

### 2.1. An Adjustable Dark-Field Acoustic-Resolution PAM (AR-PAM) Imaging System with Fiber Bundle-Based Illumination

**Figure 1:**
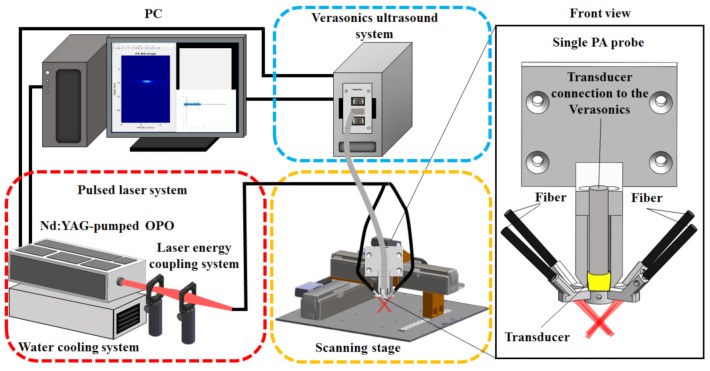
Schematic diagram of the adjustable dark-field AR dual-modality US/PA imaging system with fiber bundle-based illumination.

**Figure 2:**
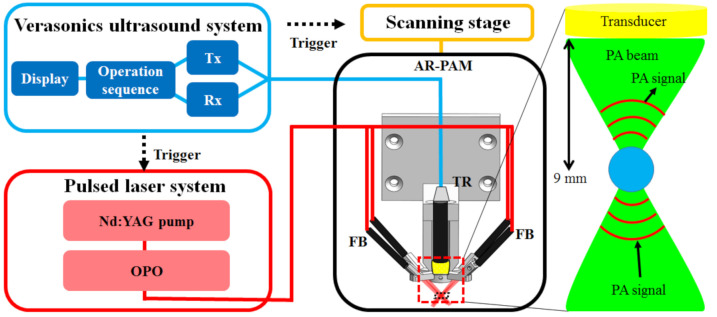
Operation sequence of the AR-PAM imaging system. A Verasonics US system sends trigger signals to an OPO laser to output a 20 Hz pulsed laser to the AR-PAM probe through a removable fiber bundle. PA signals generated by laser excitation are detected using a 50 MHz US transducer and are subsequently processed by a PC for data analysis and image processing. TR: US transducer; FB: fiber bundle; OPO: optical parametric oscillator; Tx: transmitter; Rx: receiver.

**Figure 3:**
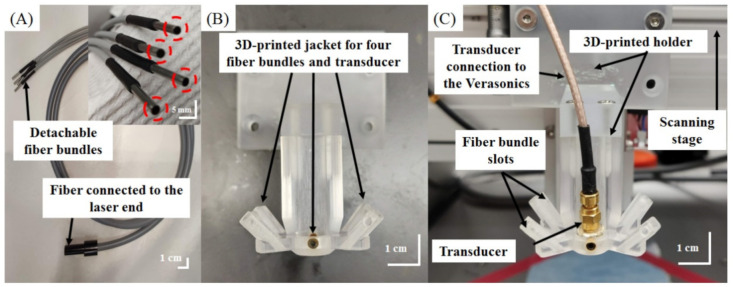
Photograph of our adjustable dark-field AR dual-modality US/PA imaging system. (**A**) Fiber bundle with one input end for connection to the laser and 4 output ends. (**B**) 3D-printed jacket for 4-fiber bundles and the transducer. (**C**) Photograph of the transducer and jacket, which were fixed to a homemade scanning stage.

The setup and operation sequence of our AR-PAM imaging system are shown in Figure 1 and Figure 2, respectively, and a detailed system block design is shown in Figure 3. For dual-modality PA/US imaging, the Verasonics high-frequency US platform (Vantage 128, Verasonics Inc., Washington, DC, USA) was employed and controlled by a custom-developed toolbox based on MATLAB^®^ (R2007a, Mathworks Inc., Natick, MA, USA). For the PA imaging mode, a trigger must be provided, which synchronizes the laser excitation and data acquisition. To efficiently collect transcranial PA signals from cortical blood vessels, the PA signals were acquired by a custom-built, large-numerical-aperture, wideband, 50 MHz US transducer [16]. This transducer had a −6 dB fractional bandwidth of 57.5%, a focal length of 9 mm, and a 6 mm active element. For excitation, the laser used was a compact Nd:YAG laser system with an integrated tunable OPO (SpitLight 600 OPO, InnoLas Laser GmbH, Krailling, Germany). Approximately 7 ns pulses at a 20 Hz repetition rate with a tunable wavelength of 680–2400 nm were generated by the OPO.

A custom-built 3D precision scanning stage (Figure 1) was constructed using two piezoelectric motors (Linear Motor Robot, Toyo Automation Co., Ltd., Tainan City, Taiwan) for movement in the x- and y-directions and a manually adjustable translation stage for movement along the *z*-axis (Sigma-koki Co., Ltd., Tokyo, Japan) [17]. Each motor had a 1 µm minimum step size, which is much smaller than the spatial resolution of the US/PA imaging system. A PC-based program controlled the precision scanning stage via a controller (PCI-1202U Driver Card, Advantech Co., Ltd., Taipei City, Taiwan) and a driver (ASD-A2R, Delta Electronics, Inc., Taipei City, Taiwan). The designed user control interface was used to easily set all parameters (i.e., speed, acceleration, and step size). An optical ruler (RH200, Renishaw Inc., Wotton-under-Edge, UK), which provides a feedback signal and is accurate up to 2 μm, was employed for positioning. The proposed US/PA system can produce A-scan, B-scan (i.e., two-dimensional, where one axis corresponds to the lateral scanning distance and the other to the imaging depth), and C-scan (i.e., 3D) images of the area of interest [11].

Figure 2 and Figure 3 show diagrams of the imaging procedure of the US/PA imaging system. The US scan was performed immediately before the PA scan so that PA images could be overlaid onto US images, and the scanning stage was used to position the transducer. Imaging was performed with the US/PA probe immersed in a water tank. For in vivo imaging, the water tank was constructed with an acoustic window by sealing a rectangular cutout at the bottom of the tank with transparent polyethylene film of 15 μm thickness [17]. US gel or gelatin pads were placed between the animal and the polyethylene film to facilitate transmission of US/PA waves. A trigger signal transmitted at every laser illumination pulse was used to synchronize the laser illumination, data acquisition, and movement of the scanning stage. After scanning, the A-line-received signal intensity was post-processed into 2D or 3D images, and US images were overlaid with PA images.

### 2.2. Design of the AR-PAM Probe—Integration of the Fiber Bundle-Based Illumination System, US Transducer, and 3D-Printed Jacket

The design of the fiber bundle illumination-based AR-PAM probe is shown in Figure 2 and Figure 3. The fiber bundle-based illumination system was custom-built (Fiberoptics Technology Inc., Pomfret Center, CT, USA), was 2 m long, and contained approximately 2071 20 μm thick multimode glass fibers with a numerical aperture (NA) of 0.25. The fiber bundle was quadrifurcated at the output end to deliver light through 4 circular bundles (diameters of 0.9 mm) (Figure 3A).

A 3D-printed jacket (2 cm × 4 cm × 4 cm) was designed to hold the 4 output ends of the fiber bundle in the configuration shown in Figure 3B and to hold the 50 MHz US transducer in the center (Figure 3C). The entire AR-PAM probe was then connected to the scanning stage via a 3D-printed holder (Figure 3B). Both the jacket and holder were first drawn using the computer-aided design (CAD) software package SolidWorks 2015 (Dassault Systèmes S.A., Velizy-Villacoublay, France). A 3D printer (Shuffle 4k, Phrozen, Inc., Hsinchu City, Taiwan) was then used to print the holder and jacket to a tolerance of 0.03 mm using an ABS-like material. The jacket was configured such that the total area from which light was delivered was less than 10 mm^2^, corresponding to the active zone of the transducer. The fiber bundles were angled to align the laser output to a depth of approximately 9 mm from the surface of the transducer.

### 2.3. Testing the Imaging Performance of the Developed Adjustable AR-PAM System

**Figure 4:**
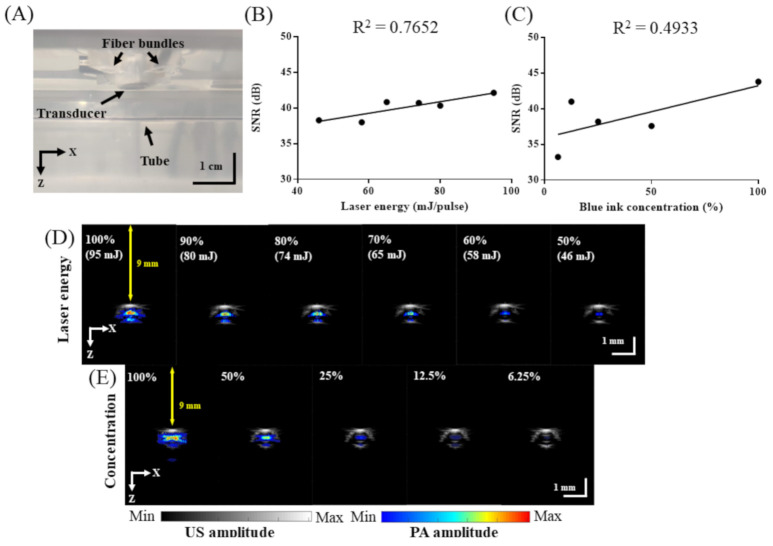
Testing of the in vitro imaging performance of our adjustable AR-PAM system. (**A**) Experimental setup in the water tank. (**B**) SNR of PA signals acquired while varying the energy of 750 nm wavelength excitation. (**C**) SNR of PA signals acquired while varying the blue ink concentration (100%, 50%, 25%, and 12.5% with saline). (**D**) Overlaid B-scan US/PA images corresponding to (**B**). (**E**) Overlaid B-scan US/PA images corresponding to (**C**).

Tube phantoms were used for in vitro testing. A transparent, low-density polyethylene tube (Scientific Commodities, Inc., Lake Havasu City, AZ, USA) with an inner diameter of 0.38 mm and an outer diameter of 1.09 mm containing blue ink (Lion Pencil Co., Ltd., New Taipei City, Taiwan) was placed in a water tank (Figure 4A) [17]. Intralipid (5%, Sigma-Aldrich, Inc., Merck, Germany) was used in the water tank to mimic optical diffusion in vivo [10]. The tube was fixed at a 9 mm depth for US and PA signal acquisition with a 750 nm excitation wavelength [17]. The laser energy (100%, 90%, 80%, 70%, 60%, and 50%) or the blue ink concentration (undiluted and diluted to 50%, 25%, 12.5%, and 6.25% with saline) was varied [17]. The laser energy was measured by an energy monitor in the OPO system.

To measure the spatial resolution, both US and PA images were acquired for a carbon fiber with a diameter of approximately 6 μm. A light-emitting diode (LED)-illuminated handheld microscope (Aca1920–155um, Basler AG, Ahrensburg, Germany) was used to confirm the diameter. A laser excitation wavelength of 750 nm was used for PA imaging. After normalization of the PA signal and plotting of the changes in the signal in the lateral and axial directions, the resolution was measured as the full-width at half-maximum (FWHM).

### 2.4. Imaging of Blue and Red Inks Using the Developed PA Spectrum Technique

Blue and red inks in tubes were used to mimic oxygenated and deoxygenated hemoglobin in blood vessels without having to set-up a more complicated flow system with oxygenation control and animal blood [32]. We tested different excitation wavelengths within the range used for in vivo imaging (700–850 nm). Blue or red ink (Lion Pencil Co., Ltd., New Taipei City, Taiwan) was first added to the tube phantom described in Section 2.3. To mimic light scattering when imaging tissue in vivo, the tube was submerged in water containing 5% Intralipid. Then, PA signals were collected and normalized by the laser power to account for fluctuations in the laser output. The normalized amplitude was plotted against the wavelength. For each excitation wavelength, 10 PA signals were collected and averaged.

### 2.5. Imaging of a Hair Phantom to Assess the 3D Imaging Capability of the AR-PAM System

A hair phantom was created by fixing 3 hairs in a water tank at different depths with overlap in the x-y plane. The tank was filled with water containing 5% Intralipid to mimic optical diffusion in vivo. A PA C-scan was acquired over an 8 mm × 8 mm region of interest (ROI) using an 800 nm laser wavelength.

### 2.6. In Vitro Test of PA Imaging Using a Chicken Breast Phantom

To test the PA imaging depth capabilities of the AR-PAM system, an oblique cut was made in chicken breast tissue, and black tape was inserted for PA contrast. The chicken breast phantom was then submerged in a water tank, and a PA B-scan was acquired at an 800 nm laser wavelength along the length of the black tape to obtain measurements at different depths.

### 2.7. In Vivo Vascular Mapping of Rat Ears and Functional Imaging of the Rat Brain with Electrical Stimulation

Rat ear and brain imaging experiments were performed on Sprague-Dawley (SD) rats (BioLASCO Taiwan Co., Ltd., Taipei City, Taiwan), which weighed 250–350 g. The experimental procedures were approved by the Institutional Animal Care and Use Committee of the National Health Research Institute (approved protocol number: NHRI-IACUC-107100-A).

For functional imaging of the S1FL motor sensory area, rats were first anesthetized with 1.5–2% isoflurane (Bowlin Biotech Corp., Taipei City, Taiwan) and then subsequently mounted on a custom-made acrylic stereotaxic head holder [18]. The skin and muscle were cut away from the skull to expose the bregma, which was used as a landmark. A high-speed drill was used to create an 8 (anterior-posterior; AP) × 6 (medial-lateral; ML) mm bilateral cranial window [18].

The AR-PAM probe was used to image brain vasculature (i.e., SSS) at bregma +1 mm, which corresponds to the primary forelimb somatosensory cortex (S1FL) area [18]. Stainless-steel needle electrodes were inserted into the left forepaws of the rats. An electrical stimulator (Model 2100, A-M Systems, Sequim, WA, USA) was used to apply a monophasic constant current at a frequency of 3 Hz [18]. The pulse duration was 0.2 ms, with an intensity of 5 mA. PA images were acquired using excitation wavelengths of 750, 800, and 850 nm [18].

### 2.8. In Vivo Functional ICG-Based Pharmacokinetic Imaging of Rat Brains

Five SD rats weighing 250–350 g were used for in vivo functional ICG-based pharmacokinetic imaging of the brain (approved protocol number: NHRI-IACUC-107100-A) [18]. After craniotomy, ICG (Sigma-Aldrich, Inc., Merck, Germany) in saline was intravenously injected at a dose of 0.25 mL/100 g of body weight. Before and after ICG injection, PA images were acquired at 810 nm for 2 and 30 min, respectively, to dynamically monitor the ICG circulation in the rat brain.

## 3. Results

### 3.1. In Vitro Performance of the Developed AR-PAM Imaging System

To assess the in vitro performance of the AR-PAM probe, blue ink-containing tubes (Figure 4A) were imaged at a fixed 9 mm depth for various laser energies and ink concentrations. The SNR increased with both the input laser pulse energy (Figure 4B) and ink concentration (Figure 4C). Overlaid US and PA images of the ink-filled tubes are shown in Figure 4D for various laser energies from 50 to 100% and in Figure 4E for various ink concentrations (100%, 50%, 25%, 12.5%, and 6.25% in saline). These results demonstrate that at 750 nm excitation, the SNR is best at 100% laser power (95 mJ) with undiluted blue ink and is acceptable down to 50% laser power (46 mJ) and with blue ink diluted to 6.25% in saline.

**Figure 5:**
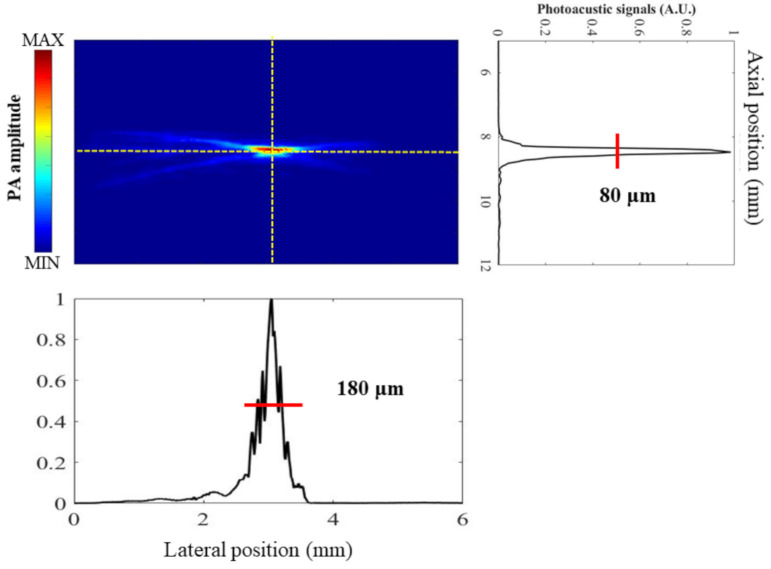
Measurement of axial and lateral resolutions using a 6 µm carbon fiber tube. An axial resolution of 80 ± 5 μm and a lateral resolution of 180 ± 32 μm were obtained.

**Figure 6:**
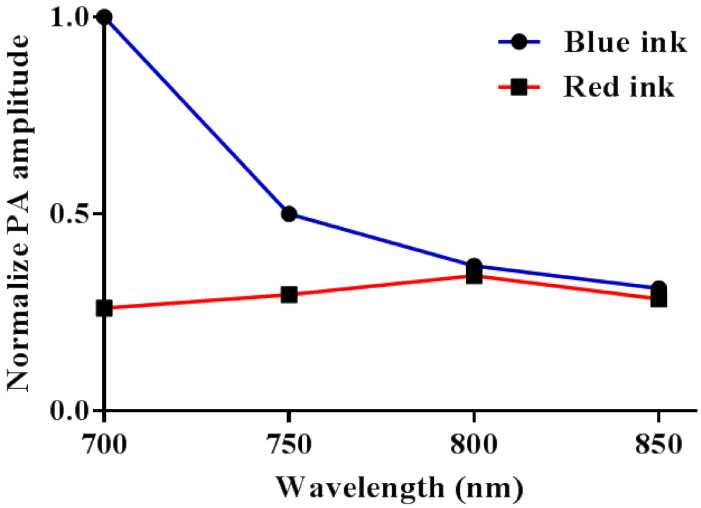
Comparison of PA signals from blue and red inks at laser excitation wavelengths of 700, 750, 800, and 850 nm. Blue and red inks can be easily distinguished when imaged at 700 nm but not at 800 nm and above.

Figure 5 shows the results of measuring the axial and lateral resolutions at a depth of 8.95 mm using a 6 μm-diameter carbon fiber. An axial resolution of 80 ± 5 μm and a lateral resolution of 180 ± 32 μm were obtained. Additionally, a comparison of PA signals from blue and red inks is shown in Figure 6. Tubes were scanned at wavelengths of 700, 750, 800, and 850 nm. The difference in PA signal amplitude between blue and red inks is maximal with an excitation wavelength of 700 nm and negligible for wavelengths of 800 nm and above (i.e., 850 nm). This result is similar to measurements of the absorption spectra of oxygenated and deoxygenated hemoglobin except that the absorption of oxygenated hemoglobin is stronger than that of deoxygenated hemoglobin at 850 nm [33]. Thus, blue and red inks in a tube phantom can be used as a simple initial model for blood vessels, but using animal blood would be a more accurate model.

### 3.2. Imaging Ink-Filled Tube, Hair, and Chicken Tissue Phantoms In Vitro

**Figure 7:**
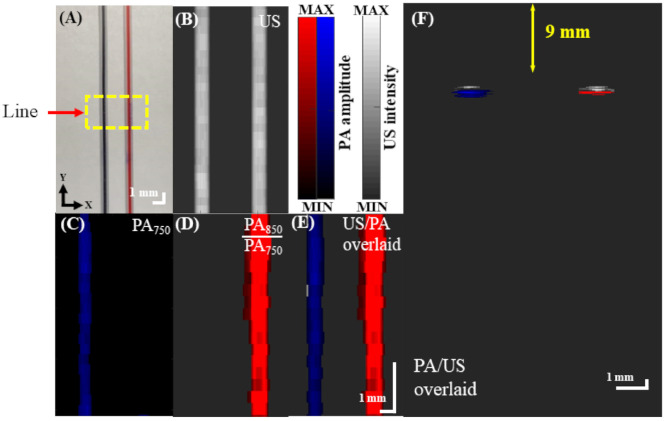
US/PA imaging of blue and red inks in the vasculature-mimicking phantom. (**A**) US/PA imaging of a rectangular region (6 × 12.695 mm^2^) indicated by yellow dashed lines of the phantom. MAP images from (**B**) US scanning of the tubes and (**C**) PA imaging of the ink-filled tubes at a 750 nm laser wavelength. (**D**) Proportional PA MAP image (PA_850_/PA_750_) of the phantom. The C-scan was acquired with a 0.1 mm step size and a 2 mm/s speed. (**E**) Combined overlaid US/PA MAP image of the ink-filled tubes. US: ultrasound; PA: photoacoustic; PA_750_: PA signal at a 750 nm excitation wavelength; PA_850_: PA signal at an 850 nm excitation wavelength; MAP: maximum amplitude projection. (**F**) Overlaid US/PA B-scan image of the ink-filled tube phantom at the position labeled “line” in (**A**).

An ink-filled tube phantom experiment was used to assess the volumetric imaging capability of our adjustable AR-PAM system [17], as illustrated in Figure 7A. As only a planar image is acquired at one depth, the compact PA probe had to be moved in the depth direction to acquire a volumetric image, which was displayed in two dimensions as a maximum amplitude projection (MAP) image. The subsequent US, PA, and overlaid US/PA MAP images of the ink-filled tube phantom are shown in Figure 7B–E. In the PA MAP images, the optical absorption characteristics of each tube were distinguishable between two excitation wavelengths (i.e., 750 and 850 nm) (Figure 7C,D) [6], which was not the case for the US MAP image (Figure 7B). The PA signal of the blue tube was dominant at 750 nm (Figure 7C), while the PA signals of both tubes were similar at 850 nm. To distinguish the red tube from the blue tube, the proportional difference (PA_Red_ = PA_850_/PA_750_) between the two excitation wavelengths was calculated for each pixel of the image (Figure 7D) [20]. In addition, the US and PA MAP images are overlaid in Figure 7E, and Figure 7F shows a PA B-scan image of the tubes at the position labeled “line” in Figure 7A. Here, the US image is represented in grayscale, whereas the PA images are represented in blue/red scale. The resulting image simultaneously visualizes the optical absorption characteristics of the ink-filled tube phantom and provides structural information. This demonstrates the feasibility of imaging oxygenated vs. deoxygenated hemoglobin in vivo by using 750 nm excitation to visualize deoxygenated blood and the PA_850_/PA_750_ signal to visualize oxygenated blood.

**Figure 8:**
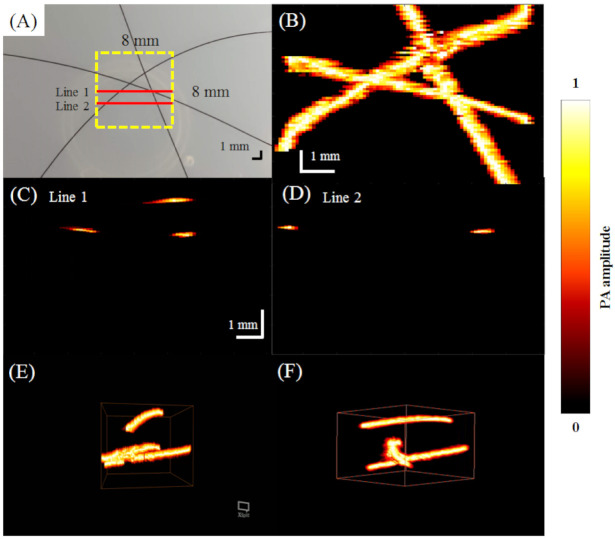
3D PA images of a tissue phantom with 3 human hairs embedded obtained using a 50 MHz transducer. (**A**) Photograph of the hair phantom created for AR-PAM. (**B**) PA C-scan image. (**C**,**D**) PA B-scan images at red Line 1 and Line 2 indicated in (**A**). (**E**,**F**) 3D PA images (Videos S1 and S2).

Figure 8A shows a stereomicroscopic image of the phantom with three hairs embedded for an 8 mm × 8 mm ROI [27]. With the overlapping hairs, the hair phantom had increased complexity compared to the tube phantom and was scanned to demonstrate the 3D imaging capabilities of the AR-PAM system. The corresponding PA MAP image of the ROI is shown in Figure 8B. Structural information of the hairs is visualized in Figure 8C,D, which show the PA B-scan images at Lines 1 and 2, respectively, depicted in red in Figure 8A. Figure 8E,F show example 3D PA C-scan images of the hair phantom. The overlapping hairs could be resolved in the z-direction.

**Figure 9:**
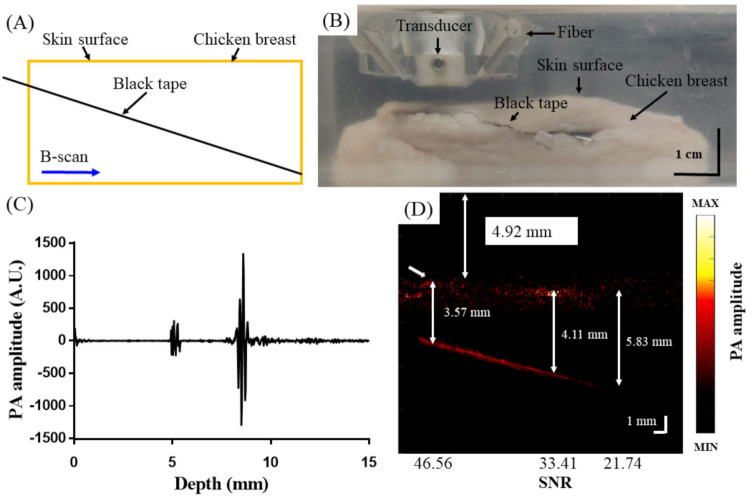
Testing the performance of the AR−PAM probe by imaging a black piece of tape obliquely inserted into chicken breast tissue. (**A**) Experimental setup; (**B**) representative image of the experimental setup; (**C**) PA A−scan signal; (**D**) PA B−scan image.

To test the tissue imaging performance of the AR-PAM probe in vitro, black tape was inserted into chicken breast tissue, as shown in the schematic in Figure 9A. A photograph of the setup is shown in Figure 9B. Figure 9C shows the PA A-scan signal and PA B-scan image (Figure 9D) obtained by the developed adjustable AR-PAM. The black tape could be visualized to a depth of 5.83 mm beneath the tissue surface. The SNRs at imaging depths of 3.57 mm, 4.11 mm, and 5.83 mm were 46.56, 33.41, and 21.74, respectively. With laser light scattering and interference from the chicken tissue, the tape could still be visualized ~9 mm from the probe surface. The results of these in vitro imaging experiments indicate that the present imaging system can visualize blood vessels with diameters of a few hundred micrometers located at least 9 mm below the surface of the transducer.

### 3.3. In Vivo Imaging of Blood Vessels in the Rat Ear

**Figure 10:**
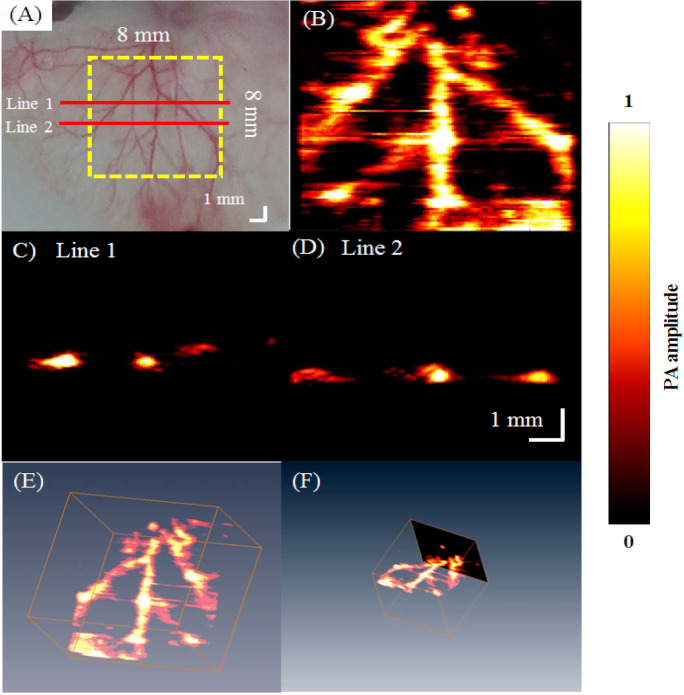
In vivo PA imaging of rat ear vasculature. (**A**) Image of the blood vessels scanned; (**B**) PA C-scan image; (**C**,**D**) PA B-scan images obtained for Lines 1 and 2 shown by the red solid lines in (**A**); (**E**,**F**) 3D PA images (Videos S3 and S4).

A photograph of the imaged area is shown in Figure 10A. With a step size of 80 μm, approximately 180 min was needed to acquire unidirectional B-scan images of the 8 mm × 8 mm area. The MAP image is shown in Figure 10B. Figure 10C,D show the PA B-scan images of Lines 1 and 2, respectively, shown in red in Figure 10A. The results of this experiment suggest that the spatial resolution and sensitivity of our developed AR-PAM are suitable for imaging blood vessels ~100 µm in diameter in vivo.

### 3.4. Evaluating Hemodynamic Changes in the SSS during Electrical Stimulation of the Left Forepaw

**Figure 11:**
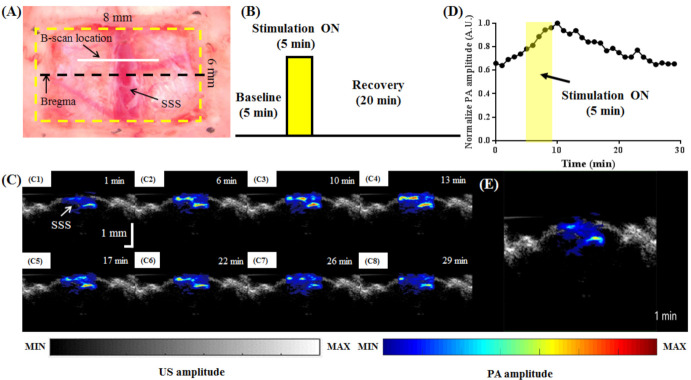
PA B-scan images of the SSS area obtained using an 800 nm excitation wavelength during electrical stimulation of the left forepaw. (**A**) Photograph of the rat brain after a craniotomy was performed for AR-PAM monitoring. (**B**) Schematic of the image acquisition timeline. (**C**) Representative US/PA-overlaid B-scan images obtained before, during, and after stimulation. (**D**) Normalized PA amplitude before, during, and after stimulation. The yellow rectangle indicates the 5 min “Stimulation ON” period. (**E**) Cerebral hemodynamics monitored in the rat brain in real time with 800 nm excitation (Video S5). The PA signal in the SSS region increased during stimulation and decreased after turning the stimulation off.

PA B-scan images of the SSS area obtained using an 800 nm excitation wavelength during electrical stimulation of the left forepaw are shown in Figure 11. After craniotomy (Figure 11A), the changes in cerebral hemodynamics induced by left-forepaw stimulation were imaged according to the schematic in Figure 11B. First, a 300 s baseline was recorded. Next, electrical stimulation was applied for 60 s. Last, a recovery period of 1200 s was recorded. The US/PA-overlaid B-scan images are shown in Figure 11C. Figure 11D shows the normalized PA amplitude at various time points over the entire signal recording period (i.e., before and during stimulation and during the recovery stage). The yellow rectangle indicates the 60 s “Stimulation ON” period. The cerebral hemodynamics in the blood vessels of the SSS were monitored in real time in a rat brain with 800 nm excitation (Video S5). The PA signal in the SSS region increased during stimulation and decreased after turning the stimulation off [31]. This is consistent with stimulation of the forepaw, which increases neural activity in the sensory motor region of the brain. Increased neural activity leads to increased blood flow to the area. Once the stimulation is turned off, blood flow returns to the baseline, as neural activity also returns to the baseline. Our AR-PAM system can detect changes in cerebral blood flow due to functional stimulation that occurs on the scale of minutes.

### 3.5. In Vivo Functional Imaging of Rat Brain Pharmacokinetics Following ICG Injection

**Figure 12:**
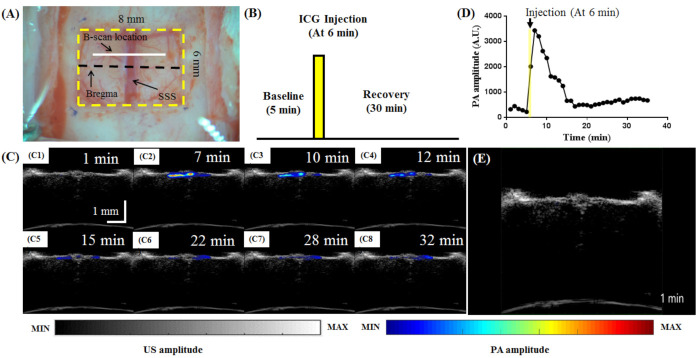
In vivo-obtained PA_810_ B-scan images of the rat brain pharmacokinetics at bregma + 1 mm following ICG injection. (**A**) Schematic showing the B-scan location. (**B**) PA image acquisition timeline at an excitation wavelength of 810 nm before, during, and after intravenous injection of ICG. (**C**) Representative US/PA overlaid B-scan images obtained before, during, and after injection of ICG. (**D**) Changes in the PA amplitude in the SSS area at different time points before, during, and after injection of ICG. The yellow rectangle indicates the 6 min mark at which ICG was injected. (**E**) Video of the changes in the PA signal (Video S6). SSS: superior sagittal sinus; ICG: indocyanine green; PA: photoacoustic.

Figure 12 shows the in vivo-obtained PA_810_ B-scan images of cerebral pharmacokinetics at bregma + 1 mm following ICG injection. A craniotomy was performed to monitor the surgical area (Figure 12A). Data were collected according to a block design paradigm (Figure 12B) involving an ICG injection. The task began with a baseline state applied for 5 min. Then, ICG was injected 1 min later (i.e., 6 min after the onset of the baseline state), after which the rat was monitored for 30 min, which included the recovery time. Representative PA B-scan images acquired before, during, and after ICG injection are shown in Figure 12C. The arrow in Figure 12D indicates the time at which ICG was injected (i.e., 6 min after the onset of the baseline period). The PA signal increased after ICG injection and subsequently decreased over time [34]. The PA signal returned to near the baseline level, as expected as ICG mainly binds to plasma proteins and does not extravasate in healthy rats with an intact blood–brain barrier [35]. A video of the changes in the PA signal is included (Video S6) to visualize the ICG brain pharmacokinetics. This result demonstrates that our AR-PAM system can be used to monitor ICG kinetics in the brain. Diseases such as tumors, stroke, or cerebral trauma can cause changes to cerebral blood flow and disruption to the blood–brain barrier. By incorporating these disease models in future studies, the comparison of ICG kinetics with those in normal controls can be explored as a disease marker.

## 4. Discussion

We developed a dual-modality, compact AR-PAM imaging system consisting of a light-adjustable fiber-bundle-based illumination system integrated with a US platform and a high-frequency transducer. Although we previously reported a PA imaging system with an array transducer [17] that could be used for small-animal whole-body imaging, the current system utilizes a high-frequency single-element transducer with increased resolution that is more suited for small-scale and shallow imaging. We first tested the AR-PAM system using red and blue inks in tubes to mimic in vivo vasculature. Although there are flaws to using red and blue inks as a model of oxygenated and deoxygenated hemoglobin due to differences in the absorption spectra, red and blue inks have been previously used with similar results [32]. Next, we imaged a hair phantom and a chicken tissue phantom to determine the limits of the AR-PAM system in 3D imaging. We could image up to ~4 mm beneath the tissue surface and could complete a C-scan of an 8 mm × 8 mm ROI within ~3 h, which is comparable to the AR-PAM system from other groups [15]. For in vivo studies, we demonstrated that our AR-PAM system could be used to image rat ear vasculature and to monitor hemodynamic changes due to neural activity induced by electrical stimulation of the forepaw. The results of the electrical stimulation experiment showed increased blood flow due to stimulation, and they agree with previous studies [16,36]. Our experiment differs from that of Ntziachristos et al. in that blood flow was measured in the SSS instead of the S1FL sensory motor region of the cortex [36]. We chose to monitor the SSS as it is easily located, while the S1FL region is more specific to stimulation [37]. We also showed that the AR-PAM system could be used to monitor the cerebral pharmacokinetics of the contrast agent ICG in real time in vivo.

As AR-PAM systems are not limited within the optical diffusion limit (~1 mm), the imaging depth is greater compared to optical-resolution PA microscopy (OR-PAM) systems [10,11]. However, the resolution is sacrificed with the gain in imaging depth. Various AR-PAM systems have been developed to improve the resolution to be comparable to OR-PAM systems. Dark-field AR-PAM systems increase resolution by using lens systems to create dark-field illumination that avoids illuminating more superficial areas of the tissue that can produce interfering signals. The configuration of the four fibers in our AR-PAM system was also designed to create a dark-field illumination effect. However, the use of fiber illumination is not as precise as the use of mirrors and lenses in traditional AR-PAM systems [15], and thus, we were unable to achieve as high a resolution. Vinneau et al. demonstrated another way to improve resolution by combining images obtained by two orthogonally oriented transducers in their dual-view AR-PAM system [38]. Omar et al. developed raster-scan optoacoustic mesoscopy systems with increased resolution using ultrawideband high-frequency ultrasound transducers and a tomographic reconstruction technique [39,40]. Another aspect of our AR-PAM system that can be improved in future iterations is the scanning speed in order to achieve real-time 3D imaging. Compared to other systems, our AR-PAM system is limited by the speed of the mechanical scanning stage and the 20 Hz laser repetition rate. Other groups have utilized microelectromechanical systems (MEMS) scanners to improve the scanning speed [41,42,43]. MEMS scanners are combined with high-repetition-rate lasers to obtain scanning speeds up to 1000 Hz for B-scans.

Although the imaging speed is slower and the resolution of our system is lower than other AR-PAM systems are, our main goal was to create an AR-PAM system with a smaller size and simple setup that does not require complicated calibration before use. With a more compact size and lighter weight, we plan to adapt our system to monitor changes in cerebral hemodynamics in awake animals as anesthesia affects hemodynamics. Collectively, this customizable US/PA imaging system can complement the existing optical imaging techniques and offers a useful tool for preclinical PA studies of smaller imaging areas that require higher resolution.

## Data Availability

Data will be provided on request through the corresponding author (L.-D.L.) of this article.

## Supplementary Materials

The following are available online at https://www.mdpi.com/article/10.3390/bios11080262/s1: Video S1 for Figure 8, Video S2 for Figure 8, Video S3 for Figure 10, Video S4 for Figure 10, Video S5 for Figure 11, and Video S6 for Figure 12.

## References

[1] Hillman E.M.C., Amoozegar C.B., Wang T., McCaslin A.F.H., Bouchard M.B., Mansfield J., Levenson R.M. (2011). In vivo optical imaging and dynamic contrast methods for biomedical research. Philos. Trans. R. Soc. A Math. Phys. Eng. Sci..

[2] Balas C. (2009). Review of biomedical optical imaging—A powerful, non-invasive, non-ionizing technology for improving in vivo diagnosis. Meas. Sci. Technol..

[3] Liao L.-D., Tsytsarev V., Delgado-Martínez I., Li M.-L., Erzurumlu R., Vipin A., Orellana J., Lin Y.-R., Lai H.-Y., Chen Y.-Y. (2013). Neurovascular coupling: In vivo optical techniques for functional brain imaging. Biomed. Eng. Online.

[4] Paddock S.W., Eliceiri K.W. (2014). Laser Scanning Confocal Microscopy: History, Applications, and Related Optical Sectioning Techniques. Methods Mol. Biol..

[5] Flores S.M., Toca-Herrera J.L. (2009). The new future of scanning probe microscopy: Combining atomic force microscopy with other surface-sensitive techniques, optical microscopy and fluorescence techniques. Nanoscale.

[6] Hoshi Y., Yamada Y. (2016). Overview of diffuse optical tomography and its clinical applications. J. Biomed. Opt..

[7] Applegate M.B., Istfan R.E., Spink S., Tank A., Roblyer D. (2020). Recent advances in high speed diffuse optical imaging in biomedicine. APL Photonics.

[8] Denk W., Strickler J.H., Webb W.W. (1990). Two-photon laser scanning fluorescence microscopy. Science.

[9] Yao J., Wang L.V. (2014). Sensitivity of photoacoustic microscopy. Photoacoustics.

[10] Wang L. (2008). Tutorial on Photoacoustic Microscopy and Computed Tomography. IEEE J. Sel. Top. Quantum Electron..

[11] Beard P. (2011). Biomedical photoacoustic imaging. Interface Focus.

[12] Wang L.V., Hu S. (2012). Photoacoustic Tomography: In Vivo Imaging from Organelles to Organs. Science.

[13] Wang S., Lin J., Wang T., Chen X., Huang P. (2016). Recent Advances in Photoacoustic Imaging for Deep-Tissue Biomedical Applications. Theranostics.

[14] Fu Q., Zhu R., Song J., Yang H., Chen X. (2018). Photoacoustic Imaging: Contrast Agents and Their Biomedical Applications. Adv. Mater..

[15] Zhang H.F., Maslov K., Stoica G., Wang L. (2006). Functional photoacoustic microscopy for high-resolution and noninvasive in vivo imaging. Nat. Biotechnol..

[16] Liao L.-D., Lin C.-T., Shih Y.-Y.I., Duong T., Lai H.-Y., Wang P.-H., Wu R., Tsang S., Chang J.-Y., Li M.-L. (2012). Transcranial Imaging of Functional Cerebral Hemodynamic Changes in Single Blood Vessels using in vivo Photoacoustic Microscopy. Br. J. Pharmacol..

[17] Leng H., Wang Y., Jhang D.-F., Chu T.-S., Tsao C.-H., Tsai C.-H., Giamundo S., Chen Y.-Y., Liao K.-W., Chuang C.-C. (2019). Characterization of a Fiber Bundle-Based Real-Time Ultrasound/Photoacoustic Imaging System and Its In Vivo Functional Imaging Applications. Micromachines.

[18] Liao L.-D., Li M.-L., Lai H.-Y., Shih Y.-Y.I., Lo Y.-C., Tsang S., Chao P.C.-P., Lin C.-T., Jaw F.-S., Chen Y.-Y. (2010). Imaging brain hemodynamic changes during rat forepaw electrical stimulation using functional photoacoustic microscopy. NeuroImage.

[19] Liao L.-D., Liu Y.-H., Lai H.-Y., Bandla A., Shih Y.-Y.I., Chen Y.-Y., Thakor N.V. (2015). Rescue of cortical neurovascular functions during the hyperacute phase of ischemia by peripheral sensory stimulation. Neurobiol. Dis..

[20] Bandla A., Liao L.-D., Chan S.J., Ling J.M., Liu Y.-H., Shih Y.-Y.I., Pan H.-C., Wong P.T.-H., Lai H.-Y., King N.K.K. (2017). Simultaneous functional photoacoustic microscopy and electrocorticography reveal the impact of rtPA on dynamic neurovascular functions after cerebral ischemia. Br. J. Pharmacol..

[21] Liu Y.-H., Liao L.-D., Tan S.S.H., Kwon K.Y., Ling J.M., Bandla A., Shih Y.-Y.I., Tan E.T.W., Li W., Ng W.H. (2015). Assessment of neurovascular dynamics during transient ischemic attack by the novel integration of micro-electrocorticography electrode array with functional photoacoustic microscopy. Neurobiol. Dis..

[22] Chuang Y.-C., Chu C.-H., Cheng S.-H., Liao L.-D., Chu T.-S., Chen N.-T., Paldino A., Hsia Y., Chen C.-T., Lo L.-W. (2020). Annealing-modulated nanoscintillators for nonconventional X-ray activation of comprehensive photodynamic effects in deep cancer theranostics. Theranostics.

[23] Sheng Y., De Liao L., Thakor N.V., Tan M.C. (2014). Nanoparticles for Molecular Imaging. J. Biomed. Nanotechnol..

[24] Li C., Yang X.-Q., An J., Cheng K., Hou X.-L., Zhang X.-S., Song X.-L., Huang K.-C., Chen W., Liu B. (2019). A near-infrared light-controlled smart nanocarrier with reversible polypeptide-engineered valve for targeted fluorescence-photoacoustic bimodal imaging-guided chemo-photothermal therapy. Theranostics.

[25] Cai X., Liu X., Liao L., Bandla A., Ling J.M., Liu Y.-H., Thakor N., Bazan G.C., Liu B. (2016). Encapsulated Conjugated Oligomer Nanoparticles for Real-Time Photoacoustic Sentinel Lymph Node Imaging and Targeted Photothermal Therapy. Small.

[26] Cai X., Bandla A., Chuan C.K., Magarajah G., Liao L.-D., Teh D.B.L., Kennedy B.K., Thakor N.V., Liu B. (2018). Identifying glioblastoma margins using dual-targeted organic nanoparticles for efficient in vivo fluorescence image-guided photothermal therapy. Mater. Horiz..

[27] Sheng Y., Liao L.-D., Bandla A., Liu Y.-H., Yuan J., Thakor N., Tan M.C. (2017). Enhanced near-infrared photoacoustic imaging of silica-coated rare-earth doped nanoparticles. Mater. Sci. Eng. C.

[28] Geng J., Liao L.-D., Qin W., Tang B.Z., Thakor N., Liu B. (2015). Fluorogens with Aggregation Induced Emission: Ideal Photoacoustic Contrast Reagents Due to Intramolecular Rotation. J. Nanosci. Nanotechnol..

[29] Razansky D., Bühler A., Ntziachristos V. (2011). Volumetric real-time multispectral optoacoustic tomography of biomarkers. Nat. Protoc..

[30] Upputuri P.K., Pramanik M. (2016). Recent advances toward preclinical and clinical translation of photoacoustic tomography: A review. J. Biomed. Opt..

[31] Grinvald A., Frostig R.D., Lieke E., Hildesheim R. (1988). Optical imaging of neuronal activity. Physiol. Rev..

[32] Kim J., Park S., Jung Y., Chang S., Park J., Zhang Y., Lovell J.F., Kim C. (2016). Programmable Real-time Clinical Photoacoustic and Ultrasound Imaging System. Sci. Rep..

[33] Lin A.J., Ponticorvo A., Konecky S.D., Cui H., Rice T.B., Choi B., Durkin A.J., Tromberg B.J. (2013). Visible spatial frequency domain imaging with a digital light microprojector. J. Biomed. Opt..

[34] Xiang L., Wang B., Ji L., Jiang H. (2013). 4-D Photoacoustic Tomography. Sci. Rep..

[35] Norat P., Soldozy S., Elsarrag M., Sokolowski J., Yaǧmurlu K., Park M.S., Tvrdik P., Kalani M.Y.S. (2019). Application of Indocyanine Green Videoangiography in Aneurysm Surgery: Evidence, Techniques, Practical Tips. Front. Surg..

[36] Ovsepian S.V., Jiang Y., Sardella T.C., Malekzadeh-Najafabadi J., Burton N.C., Yu X., Ntziachristos V. (2020). Visualizing cortical response to optogenetic stimulation and sensory inputs using multispectral handheld optoacoustic imaging. Photoacoustics.

[37] Roston S. (1967). The blood flow of the brain. Bull. Math. Biol..

[38] Vienneau E., Liu W., Yao J. (2018). Dual-view acoustic-resolution photoacoustic microscopy with enhanced resolution isotropy. Opt. Lett..

[39] Omar M., Gateau J., Ntziachristos V. (2013). Raster-scan optoacoustic mesoscopy in the 25–125 MHz range. Opt. Lett..

[40] Omar M., Soliman D., Gateau J., Ntziachristos V. (2014). Ultrawideband reflection-mode optoacoustic mesoscopy. Opt. Lett..

[41] Moothanchery M., Dev K., Balasundaram G., Bi R., Olivo M. (2019). Acoustic resolution photoacoustic microscopy based on microelectromechanical systems scanner. J. Biophotonics.

[42] Kim J.Y., Lee C., Park K., Lim G., Kim C. (2015). Fast optical-resolution photoacoustic microscopy using a 2-axis water-proofing MEMS scanner. Sci. Rep..

[43] Yao J., Wang L., Yang J.-M., Gao L.S., Maslov K., Wang L., Huang C.-H., Zou J. (2012). Wide-field fast-scanning photoacoustic microscopy based on a water-immersible MEMS scanning mirror. J. Biomed. Opt..

